# First-Principles Insights into Recently Synthesized *α*′–B_8_H_4_ Hydrogenated Borophene: A Stable Semiconducting Monolayer for UV Photodetection and Optoelectronic Applications

**DOI:** 10.3390/ma19050907

**Published:** 2026-02-27

**Authors:** Bohayra Mortazavi, Masoud Shahrokhi, Fazel Shojaei, Xiaoying Zhuang

**Affiliations:** 1 Department of Mathematics and Physics, Leibniz Universität Hannover, Welfengarten 1A, 30167 Hannover, Germany; 2 Cluster of Excellence PhoenixD (Photonics, Optics, and Engineering–Innovation Across Disciplines), Gottfried Wilhelm Leibniz Universität Hannover, Welfengarten 1A, 30167 Hannover, Germany; 3 Department of Physics, Faculty of Science, University of Ostrava, 30. dubna 22, 701 03 Ostrava, Czech Republic; shahrokhimasoud37@gmail.com; 4 Department of Chemistry, Faculty of Nano and Bioscience and Technology, Persian Gulf University, Bushehr 75169, Iran; fshojaei@pgu.ac.ir

**Keywords:** hydrogenated borophene, 2D semiconductor, optoelectronics, mechanical, thermal conductivity

## Abstract

While diverse previously fabricated pristine and hydrogenated borophene lattices have been characterized predominantly by their metallic nature, a recent experimental breakthrough has introduced α′–B_8_H_4_, a semiconducting hydrogenated borophene phase, opening new avenues for boron-based nanoelectronics. Spurred by this breakthrough, herein we utilize a comprehensive first-principles framework to investigate the critical properties of α′–B_8_H_4_ monolayer. Stability analyses confirm the considerable dynamical and thermal robustness of the α′–B_8_H_4_ monolayer. Calculations using hybrid functionals show that suspended single-layer α′–B_8_H_4_ exhibits an indirect semiconducting behavior, with band gaps of 2.06 eV and 2.45 eV predicted by HSE06 and PBE0, respectively. Optical response calculations reveal strong in-plane absorbance in the UV region, with the first notable peak at ~3.65 eV and the main peak occurring between 4.20 and 4.45 eV, both of which are clearly within the ultraviolet range. Mechanical analysis reveals that α′–B_8_H_4_ exhibits decent in-plane strength (>10 N/m), while phononic transport calculations yield a moderately low room-temperature lattice thermal conductivity of ~20 W/m·K, both displaying slight anisotropic behavior. These results provide a comprehensive first-principles characterization of the α′–B_8_H_4_ monolayer, highlighting the rare emergence of semiconducting behavior in borophene derivatives and underscoring its potential for UV optoelectronics and nanoscale device applications.

## 1. Introduction

The emergence of two-dimensional (2D) materials has fundamentally redefined the landscape of modern condensed matter physics and materials science. Since the isolation of graphene [1,2,3] over two decades ago, the scientific community has sought to harness the extraordinary electronic, optical, and mechanical phenomena that arise at the atomic limit. These materials represent the frontier of a paradigm shift in technology, promising to overcome the inherent physical and thermal bottlenecks of traditional bulk silicon-based semiconductors. Monoelemental 2D materials, such as graphene, silicene [4], germanene, and stanene [5], phosphorene [6,7], borophene [8,9], and goldene [10], have redefined the field of nanotechnology, presenting a unique suite of characteristics that pave the way for cross-disciplinary innovations and high-performance devices. Among these, borophene stands out due to its rich structural polymorphism, lightweight nature, and outstanding mechanical strength and flexibility, as well as its exceptional performance in diverse energy-related applications [11,12,13,14]. To date, several pristine borophene allotropes [8,9,15,16,17] have been successfully synthesized, and hydrogenation has further expanded the accessible structural landscape [15,16,18]. Despite these advances, a persistent limitation has characterized the borophene family: experimentally realized pristine borophenes and most hydrogenated borophene phases exhibit metallic electronic behavior. This dominant metallicity has significantly restricted their integration into semiconductor-based device architectures, where a finite band gap is essential for transistor switching, photodetection, and optoelectronic functionality.

The search for semiconducting boron materials has long been an active research area, showing that a band gap can be achieved experimentally through hydrogen functionalization. An important step forward came in 2020 with the fabrication of α′–4H borophene [15], an ultrastable crystalline semiconducting monolayer with a reported band gap of 2.48 eV. Nearly five years later, building on this progress, a recent experimental study by Xu et al. [19] realized α′–B_8_H_4_ phase. Unlike the earlier α′–4H flat-like structure, α′–B_8_H_4_ shows a complex corrugated structural reconstruction caused by specific bonding patterns, further expanding the growing family of semiconducting boron allotropes. Motivated by the experimental work of Xu et al. [19], we use first-principles calculations for the first time to study the key physical properties of the suspended α′–B_8_H_4_ monolayer. We find that this phase is highly stable and exhibits indirect semiconducting behavior, with a band gap of 2.45 eV, in good agreement with experimental measurements [19]. The optical response shows strong in-plane light absorbance in the UV region, with relatively small anisotropy, highlighting its potential for ultraviolet optoelectronic applications. In addition, the material has strong mechanical stability and moderate thermal conductivity. Overall, the α′–B_8_H_4_ nanosheet appears to be a very promising material for next-generation optoelectronic devices that require both good thermal transport and mechanical strength.

## 2. Computational Methods

In this work, first-principles density functional theory (DFT) calculations were performed using the Vienna Ab initio Simulation Package (VASP) [20,21]. The generalized gradient approximation (GGA) with the Perdew–Burke–Ernzerhof (PBE) functional was used, with a plane-wave cutoff energy of 400 eV. The convergence criteria for self-consistent loop calculations were set to 10^−6^ eV for structural optimization and 10^−7^ eV for electronic and optical property calculations. Atomic positions and lattice parameters were optimized using the conjugate gradient method until the Hellmann–Feynman forces on each atom were smaller than 10^−3^ eV/Å using a fine 13 × 13 × 1 K-point Monkhorst Pack mesh [22]. A vacuum space of over 15 Å along the out-of-plane direction was introduced to avoid interactions between periodic layers. Spin-polarized test calculations were performed, and since no magnetic moment was obtained, all reported results correspond to non-spin-polarized calculations. To eliminate artificial electrostatic interactions arising from the periodic slab geometry, dipole corrections were applied along the out-of-plane direction in all calculations. The electronic properties were further investigated using the range-separated hybrid Heyd–Scuseria–Ernzerhof (HSE06) functional with default parameters (including 25% exact exchange and a screening parameter of 0.2 Å^−1^) [23], as well as the PBE0 hybrid functional incorporating the standard 25% Hartree–Fock exact exchange [24], as implemented in VASP. Based on systematic convergence tests, an 8 × 8 × 1 Monkhorst–Pack k-point mesh and a 450 eV plane-wave cutoff were identified as providing well-converged results. Since PBE0 provides a more reliable description of the electronic structure of the studied monolayer, the optical properties were derived from the frequency-dependent dielectric function calculated within the random phase approximation (RPA) based on the PBE0 functional, using a denser 12 × 12 × 1 k-point grid. Since the dielectric function was computed within the independent-particle RPA, electron–hole interactions are not explicitly included, and consequently, excitonic contributions to the optical spectra are not considered. Spin–orbit coupling (SOC) was not included in the calculations, as the system consists of light elements (B and H) for which relativistic effects are negligible and are not expected to significantly affect the electronic or optical properties.

Density functional perturbation theory (DFPT) calculations were performed using VASP to obtain phonon dispersion relations with the PHONOPY package [25], based on a 4 × 4 × 1 supercell containing 192 atoms. To substantially accelerate the calculation of lattice thermal conductivity [26,27,28,29], the moment tensor potential (MTP) [30] formalism was used. The training dataset for the MTP was generated from ab initio molecular dynamics (AIMD) simulations of the stress-free structure and two biaxially strained cases (−5% and +5%). These simulations were carried out using a 3 × 3 × 1 supercell (106 atoms) over a temperature range from 1 to 1000 K for 500 time steps. The AIMD simulation protocol remains consistent with the optimized methodology detailed in our previous studies [27,31], using a 1 fs time step, the NVT ensemble, and a 2 × 2 × 1 k-point mesh. Half of the AIMD dataset (750 configurations) was used to train the MTP. A cutoff radius of 4.0 Å was employed in the training of the MTP because the lattice dynamics of hydrogenated borophene are dominated by short-range covalent interactions. The phonon dispersion was then calculated using the trained MTP together with the small-displacement method implemented in PHONOPY, again using a 4 × 4 × 1 supercell, consistent with our earlier study [31]. Third-order anharmonic interatomic force constants were obtained from 4 × 4 × 1 supercells, including interactions up to the seventh nearest neighbors, as established in our previous work [27]. Due to the low symmetry of the system, this step required 2943 single-point force calculations for large 192-atom structures. Such calculations would be extremely demanding with direct DFT, whereas with the MTP they can be completed within minutes on a single CPU without MPI. The lattice thermal conductivity was determined by solving the phonon Boltzmann transport equation (BTE) within the relaxation time approximation using the ShengBTE [32] package. Isotope scattering was included to represent heat transport in natural samples. Thermal stability was further analyzed through 10 ps AIMD trajectories at 500 and 1000 K. For these extended simulations, more moderate computational settings, including default energy cutoffs, were adopted to facilitate longer timescales. Atomic structures were visualized using the VESTA [33] package and VASPKIT [34] was employed to find high-symmetry points of the Brillouin zone.

## 3. Results and Discussions

**Figure 1:**
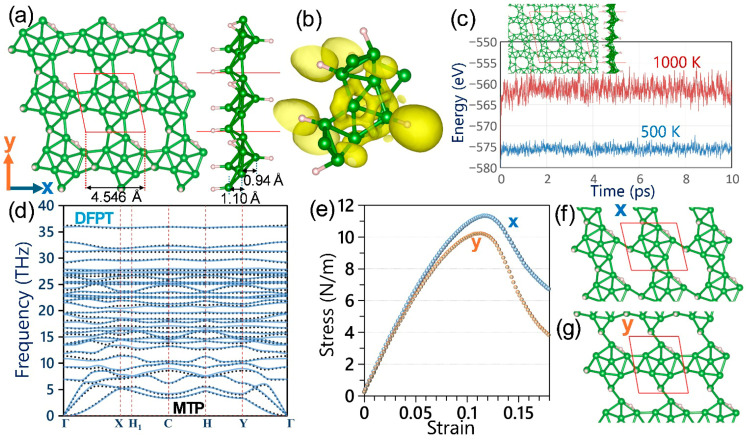
Structural, thermal, vibrational, and mechanical properties of the suspended α′–B_8_H_4_ monolayer. (**a**) Top and side views of the optimized atomic structure, showing the corrugated geometry and characteristic atomic distances. (**b**) Electron localization function (ELF) with an isosurface value of 0.75, illustrating covalent B–B bonding and charge localization near H atoms. (**c**) AIMD results for the evolution of potential energy at 500 and 1000 K; the inset shows the top and side views of the atomic configuration at 1000 K at the end of the 10 ps simulation. (**d**) Phonon dispersion obtained from DFPT (continuous lines) compared with the trained MTP (dashed lines). (**e**) Uniaxial stress–strain curves along the x and y directions, indicating slight mechanical anisotropy. (**f**,**g**) Atomic configurations near fracture under tensile strain along the x and y directions.

We first examine the structural features of the α′–B_8_H_4_ monolayer, as shown in Figure 1a. From the top view, the structure can be described as pentagon-like cone-shaped boron clusters formed by five triangles, which are linked through additional three triangular boron units. Compared with previously synthesized pristine and hydrogenated borophene sheets [15,16,18], hydrogenation in the α′–B_8_H_4_ monolayer leads to a more complex and corrugated atomic structure. The calculated lattice constant is 4.546 Å, which agrees closely with the value of 4.54 Å [19] reported in first-principles data of the original experimental study [19]. The side view in Figure 1a also shows the buckled nature of the layer, and the measured B–B bond distances match closely with reported data in the experimental study [19]. To better understand the bonding, the electron localization function (ELF) [35] is shown in Figure 1b with an isosurface value of 0.75. Strong electron localization appears between boron atoms, indicating covalent B–B bonding. Noticeable localization is also found near hydrogen atoms, confirming their participation in bonding and charge redistribution within the α′–B_8_H_4_ monolayer. Thermal stability is evaluated using AIMD at 500 K and 1000 K (Figure 1c). The total energy fluctuates only slightly with time at both temperatures, showing no structural collapse. The inset snapshots (top and side views) confirm that at 1000 K, the atomic framework of the α′–B_8_H_4_ monolayer remains intact, proving strong thermal stability.

The phonon dispersion obtained from DFPT is presented in Figure 1d. As expected for 2D materials, three acoustic branches originate from the Γ point: two in-plane modes with linear dispersion and one out-of-plane mode with quadratic behavior [36]. No imaginary frequencies are observed, confirming dynamical stability. Due to the presence of hydrogen atoms, additional high-frequency rather flat modes appear up to around 78 THz. As can also be observed, optical branches above 10 THz show relatively flat dispersions, indicating low phonon group velocity [37,38]. Frequent band crossings are also visible, which enhance phonon scattering and can reduce phonon lifetimes. We also verified the accuracy of our trained MTPs by comparing them with the DFPT results shown by the dashed lines in Figure 1d. As can be seen, the MTP predictions match the DFPT data almost perfectly. To quantitatively assess the agreement between the MTP and DFPT phonon dispersions, we calculated the root-mean-square error (RMSE) of the phonon frequencies across the Brillouin zone. The RMSE values are 0.10, 0.18, and 0.15 THz for the first three acoustic branches, respectively, and 0.21 THz for the full spectrum, confirming the strong overall agreement between the two approaches. This demonstrates that our classical model can accurately capture complex lattice dynamics at negligible cost, making it a powerful tool for large-scale computations. Mechanical properties under uniaxial tensile loading are shown in Figure 1e. During loading, the lattice size perpendicular to the strain direction was relaxed to maintain nearly zero transverse stress [39,40,41]. The α′–B_8_H_4_ monolayer shows slightly higher tensile strength along the x direction, and a steeper initial slope, indicating slight mechanical anisotropy. Along the x(y) directions, the predicted elastic modulus and tensile strength values are 144 (135) N/m and 11.3 (10.2) N/m, respectively, indicating approximately 10% anisotropy. Figure 1f,g illustrate the failure process under strain. Fracture initiates in the connecting boron triangles, particularly between hydrogenated boron atoms bonded to neighboring hydrogen-free boron atoms of the pentagonal core units. This shows that these bridge regions are the mechanically weaker parts of the α′–B_8_H_4_ monolayer.

**Figure 2:**
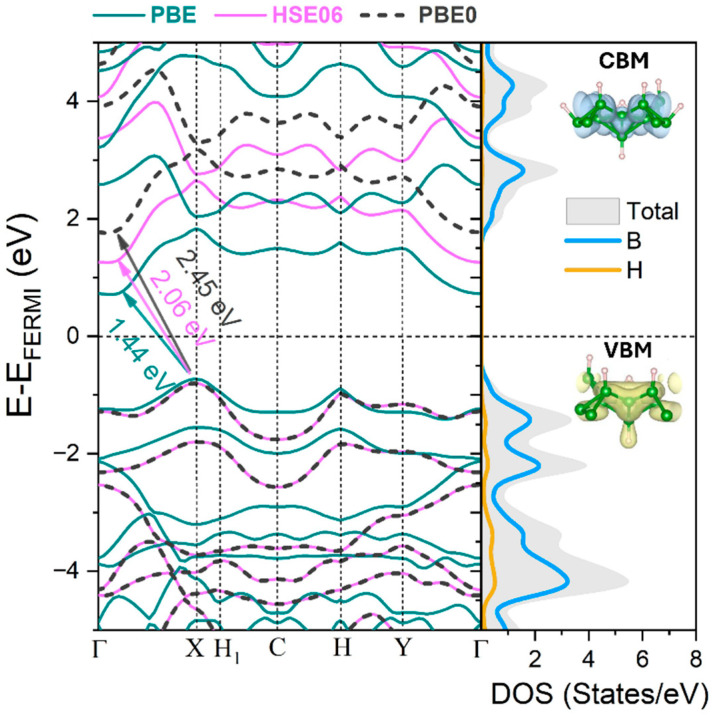
Band structures of the α′–B_8_H_4_ monolayer computed using PBE, HSE06, and PBE0 functionals, together with the total and projected density of states (DOS and PDOS) derived from PBE0 near the Fermi level. Partial charge densities of the VBM (yellow isosurfaces) and CBM (blue isosurfaces) are also shown.

We next examine the electronic and optical properties of the α′–B_8_H_4_ monolayer. The electronic band structure along high-symmetry directions in the Brillouin zone, together with the total and partial densities of states (DOS and PDOS), were computed using the PBE, HSE06, and PBE0 hybrid functionals, as shown in Figure 2. The results indicate that α′–B_8_H_4_ exhibits an indirect band gap, with the valence band maximum (VBM) located along the X direction and the conduction band minimum (CBM) along the Γ–X path for all three functionals. The corresponding band gaps are 1.44 eV, 2.06 eV, and 2.45 eV for PBE, HSE06, and PBE0, respectively, in close agreement with previous theoretical reports (1.44 eV, 2.03 eV, 2.54 eV) [19]. The experimentally measured band gap of the α′–B_8_H_4_ monolayer is approximately 2.52 eV [19], which closely matches our PBE0 result, highlighting the importance of using advanced hybrid functionals to accurately describe the electronic structure of this system. In particular, the PBE0 functional has been demonstrated to predict band gaps in borophene-based materials reliably, often providing results in closer agreement with experiment [42]. The PDOS and the partial charge density distributions of the VBM and CBM reveal that the VBM is predominantly derived from the B *p* orbitals, with a minor contribution from the H *s* states. In contrast, the CBM is mainly composed of B *p* orbitals, indicating that boron atoms play a dominant role in governing the frontier electronic states of the α′–B_8_H_4_ monolayer.

**Figure 3:**
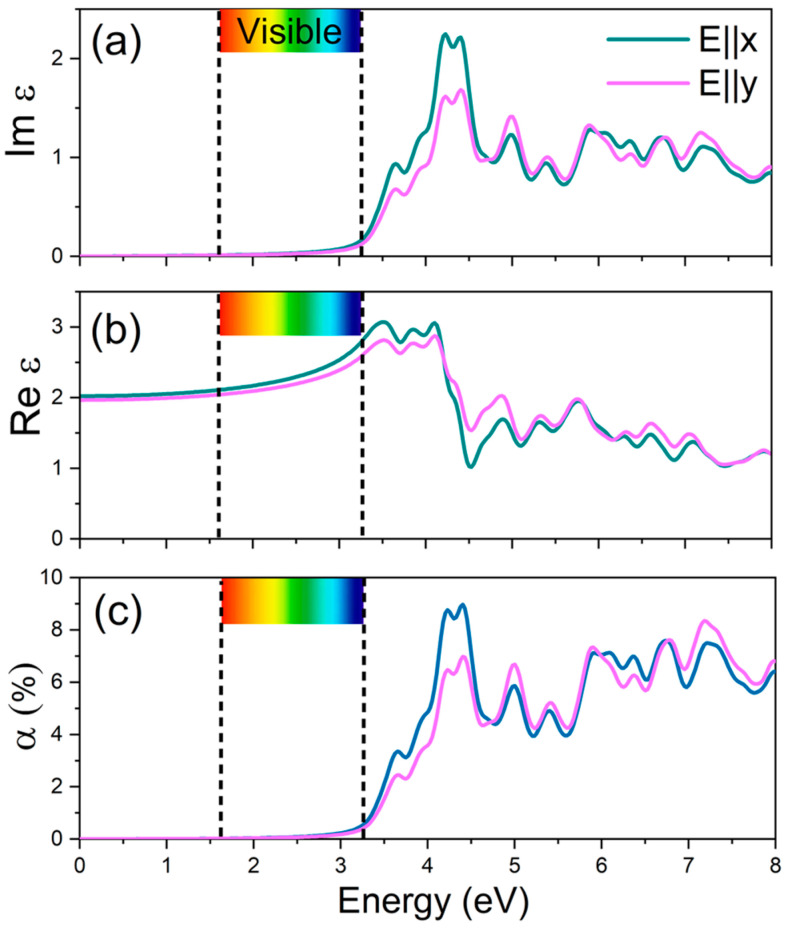
(**a**) Real and (**b**) imaginary components of the frequency-dependent dielectric function, together with (**c**) the optical absorbance (α) of the suspended α′–B_8_H_4_ monolayer, computed using the PBE0 hybrid functional. Results are presented for in-plane light polarizations along the x (E‖x) and y (E‖y) directions.

We next examine the optical properties of the α′–B_8_H_4_ monolayer by computing its dielectric function and optical absorbance within the independent-particle RPA framework, based on the PBE0 hybrid functional, which provides a band gap in close agreement with experimental measurements. Due to the previously observed intrinsic in-plane structural anisotropy along the *x* and *y* directions, the α′–B_8_H_4_ monolayer exhibits a weak polarization-dependent optical anisotropy. Owing to the pronounced depolarization effect inherent to two-dimensional systems under out-of-plane light polarization (*E*‖*z*), only the in-plane optical responses are considered in this work. The real and imaginary parts of the dielectric function, Re ε(ω) and Im ε(ω), were calculated as functions of photon energy for light polarized along the *x* (*E*‖*x*) and *y* (*E*‖*y*) directions, and the corresponding results are presented in Figure 3. The imaginary part of the dielectric function, Im ε(ω) (Figure 3a), shows its first peak feature at approximately 3.65 eV in the UV region for both in-plane polarizations. The most intense peak occurs between 4.20 and 4.45 eV, also within the ultraviolet range. As shown in Figure 3b, the static dielectric constant, Re ε(0), is calculated to be 2.02 and 1.97 for the *x*- and *y*-polarized directions, respectively, further confirming the semiconducting nature of the α′–B_8_H_4_ monolayer. The real part of the dielectric function reaches its maximum value at an energy of approximately 3.50 eV, while a pronounced minimum occurs near 4.53 eV, reflecting strong interband transition effects in this energy range. The optical absorbance (α) of the α′–B_8_H_4_ monolayer for in-plane light polarizations is presented in Figure 3c as a function of photon energy. The first absorbance peak for both x- and y-polarized light occurs at ~3.65 eV, corresponding to the UV region, while the strongest peak appears between 4.20 and 4.45 eV in the UV range, in agreement with the Im ε(ω) analysis. These optical properties, particularly the strong ultraviolet absorption, suggest that the α′–B_8_H_4_ monolayer could be a promising candidate for applications in UV photodetectors, optoelectronic devices, and ultraviolet light-harvesting technologies.

**Figure 4:**
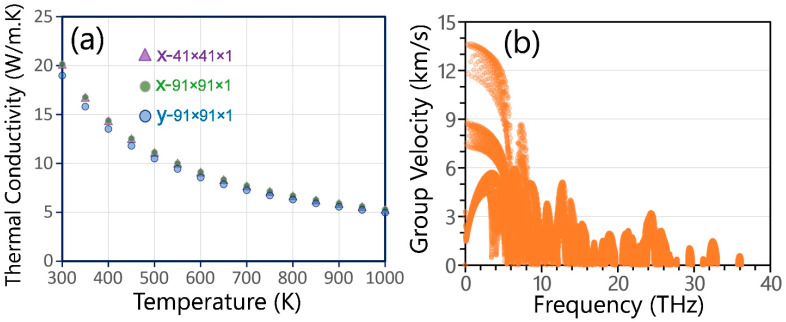
(**a**) Lattice thermal conductivity along the x and y directions as a function of temperature; calculated using 41 × 41 × 1 and 91 × 91 × 1 q-point meshes. (**b**) Phonon group velocity of the α′–B_8_H_4_ monolayer.

We finally investigate phononic thermal transport in the α′–B_8_H_4_ monolayer. While the definition of thickness for 2D materials can be controversial, we have assumed a thickness of 6.82 Å for the α′–B_8_H_4_ monolayer in this study, calculated based on the maximum vertical distance between the boundary hydrogen atoms plus their Van der Waals diameter (2.4 Å). Figure 4a shows the calculated lattice thermal conductivity along the x and y directions. To verify convergence with respect to the q-point mesh, results obtained using a 41 × 41 × 1 grid were compared with those from a denser 91 × 91 × 1 grid, demonstrating well-converged values. At room temperature (300 K), the thermal conductivity is predicted to be 20 W/m·K along x and 19 W/m·K along y. Consistent with the trends observed in the elastic and optical properties, the thermal conductivity along x is slightly higher than along the y direction, representing a weak in-plane anisotropy of approximately 5%. This directional difference can be explained by the phonon dispersion shown earlier in Figure 1d. The acoustic branches display slightly wider dispersion along the Γ–X path than along Γ–Y, which leads to slightly higher phonon group velocities in the x direction. The contributions of different phonon branches to heat transport were also analyzed. The out-of-plane acoustic (ZA) mode contributes about 13%, while the transverse acoustic (TA) and longitudinal acoustic (LA) modes contribute approximately 36% and 42%, respectively. In total, acoustic phonons account for about 91% of the lattice thermal conductivity. This behavior agrees well with graphene [43,44,45] and other two-dimensional materials, where low-frequency acoustic modes dominate heat conduction. Figure 4b presents the phonon group velocities. The LA and TA modes show slightly anisotropic starting velocities in the ranges of about 12–14 km/s and 7–9 km/s, respectively, with higher values along the x direction, which explains the slightly higher thermal conductivity along this direction. The temperature dependence of the lattice thermal conductivity follows approximately κ ∝ T^β^, where β ≈ −1.12 over the studied temperature range. This value is considerably lower than that predicted for graphene, −1.32 by Lindsay et al. [46] and −1.34 by Fugallo et al. [47]. While the proximity of β to ≈−1.0 indicates that three-phonon Umklapp scattering is the primary source of thermal resistance, the slight deviation suggests a contribution from higher-order anharmonicity or thermal expansion effects. Consequently, while the inclusion of four-phonon scattering may further reduce the thermal conductivity to values below 20 W/mK, it is not expected to lead to a substantial decrease beyond that threshold. This expectation is particularly reasonable because the relaxation time approximation herein used typically yields lower estimate of the thermal conductivity compared to the full iterative solution of the Boltzmann transport equation. Nonetheless, the precise quantification of higher-order scattering effects remains an important topic for future studies on the thermal transport of the α′–B_8_H_4_ monolayer. To better contextualize the predicted room-temperature thermal conductivity (~20 W/m·K), we note that this value is substantially lower than that of graphene (≈3000–5000 W/m·K [48,49]) and also below the typical value reported for monolayer MoS_2_ (≈35 W/m·K [50]). Consequently, the thermal transport in α′–B_8_H_4_ can be considered moderately low, indicating that it is unlikely to present significant thermal management challenges in optoelectronic applications.

## 4. Concluding Remarks

Motivated by the recent experimental realization of α′–B_8_H_4_ hydrogenated borophene [19], we carried out comprehensive first-principles calculations to explore the fundamental physical properties of the free-standing α′–B_8_H_4_ monolayer. Our results reveal that the material is dynamically stable and thermally resilient. Mechanically, the α′–B_8_H_4_ monolayer exhibits robust structural integrity with moderate in-plane anisotropy. The elastic modulus along the x and y directions is predicted to be 144 and 135 N/m, respectively, while the corresponding tensile strengths are 11.3 and 10.2 N/m. The α′–B_8_H_4_ monolayer is characterized as an indirect semiconductor with a PBE0 band gap of 2.45 eV, in close agreement with experimental measurements [19]. Its strong in-plane optical absorbance in the UV region, combined with low optical anisotropy, underscores its potential for applications in UV photodetectors and optoelectronic devices. At room temperature, the lattice thermal conductivity is predicted to be 20 W/m·K and 19 W/m·K along the x and y directions, respectively, indicating slightly anisotropic heat transport. Overall, the present first-principles study provides a comprehensive understanding of the structural stability, semiconducting behavior, optical response, mechanical resilience, and thermal transport properties of the α′–B_8_H_4_ monolayer, offering valuable insights for the design of novel optoelectronic devices.

## Data Availability

Please find: https://doi.org/10.17632/dynykhc9zy for the related data for this work. Additional data presented in this study are available on request from the first author.
